# Concomitant metastatic head-and-neck cancer and pancreatic cancer assessed by αvβ6-integrin PET/CT using ^68^Ga-Trivehexin: incidental detection of a brain metastasis

**DOI:** 10.1007/s00259-024-06750-6

**Published:** 2024-05-21

**Authors:** Jana Rehm, Robert Winzer, Johannes Notni, Sebastian Hempel, Marius Distler, Gunnar Folprecht, Jörg Kotzerke

**Affiliations:** 1 Department of Nuclear Medicine, Faculty of Medicine and University Hospital Carl Gustav Carus, Technische Universität Dresden, Dresden, Germany; 2 TRIMT GmbH, Radeberg, Germany; 3 Department of Visceral, Thoracic and Vascular Surgery, Faculty of Medicine and University Hospital Carl Gustav Carus, Technische Universität Dresden, Dresden, Germany; 4 Medical Clinic I, Faculty of Medicine and University Hospital Carl Gustav Carus, Technische Universität Dresden, Dresden, Germany

## Abstract

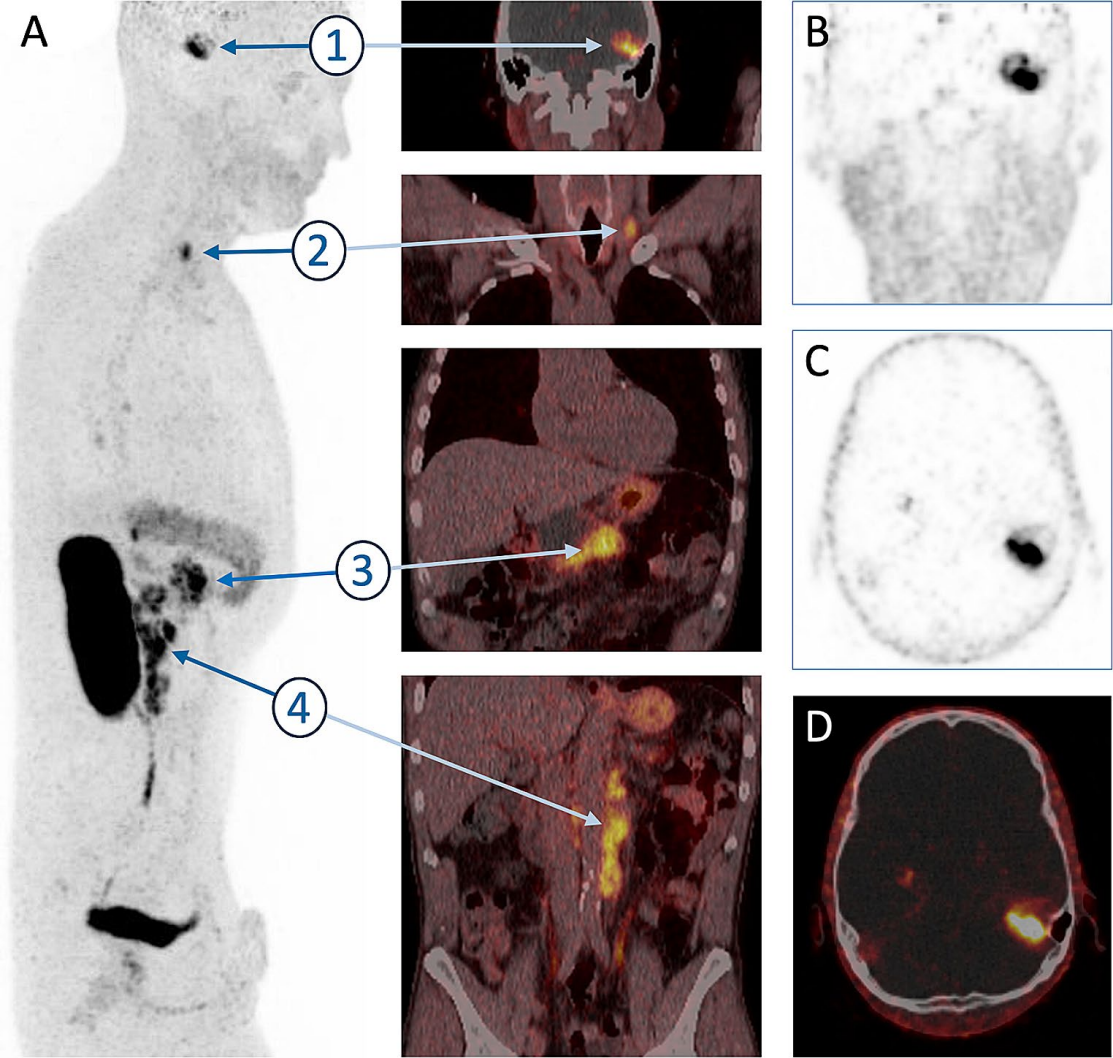

^68^Ga-Trivehexin is an investigative PET radiopharmaceutical targeting the “cancer integrin” αvβ6, a tumor cell biomarker which is highly expressed by various carcinomas [1]. Feasibility of targeting αvβ6-integrin for cancer imaging has been demonstrated in preclinical and first clinical applications [2, 3, 4, 5], warranting a clinical study of ^68^Ga-Trivehexin (NCT05799274).

The image shows a ^68^Ga-Trivehexin PET/CT (146 MBq, 60 min p.i. static scan, 5 bed positions) of a male patient (50 y, 69 kg), acquired as part of primary staging before therapy planning of a histologically confirmed pancreatic ductal adenocarcinoma (PDAC) in the pancreatic corpus. Furthermore, a tonsillar carcinoma, metastasized to the right cervical side with subsequent surgical resection and chemo-radiotherapy, was known. The lateral MIP (scaled to SUV 12) and corresponding coronal fusion (**A**) showed intense uptakes in the cerebellopontine angle (**1**, SUV_max_ 18.5) corresponding to a brain metastasis; in a supraclavicular metastasis (**2**, SUV_max_ 12.7); in the pancreatic corpus (**3**, SUV_max_ 17.0); and in retrocrural and retroperitoneal PDAC metastases (**4**, SUV_max_ 13.3). **B**, **C**, and **D** show coronal and transversal PET slices and fusion, respectively, of the cerebellopontine metastasis, which was subsequently confirmed by MRI (not shown). Left tumor exstirpation yielded a specimen for histological classification of the brain metastasis as squamous cell carcinoma (SCC) with basaloid growth, p40 and p63 positive. Cranial and supraclavicular metastases were thus assigned to tonsillar carcinoma without association to HPV according to immunohistochemistry. Altogether, ^68^Ga-Trivehexin PET/CT revealed two αvβ6-integrin expressing tumors, PDAC and SCC, and furthermore a metastasis of the latter in the brain, which is obviously not easily possible with [^18^F]FDG.

## Data Availability

The datasets used and/or analysed during the current study are available from the corresponding author on reasonable request.
